# Some residents drop out of specialty training. How important is prior clinical experience? A survey among residents in the Netherlands

**DOI:** 10.3205/zma001587

**Published:** 2023-02-15

**Authors:** Sophie J. Querido, Marlies E. J. de Rond, Lode Wigersma, Ole ten Cate

**Affiliations:** 1 University Medical Center Utrecht, Center for Research and Development of Education, Utrecht, The Netherlands; 2 Central Board for Specialty training in Elderly Care Medicine in the Netherlands (SOON), Utrecht, The Netherlands; 3 Royal Dutch Medical Association (KNMG), Utrecht, The Netherlands; 4 Dutch Association of Public Health Physicians (VAV), Utrecht, The Netherlands

**Keywords:** drop-out, residency, career choice, survey

## Abstract

**Objectives:**

The drop-out rate among residents across all medical specialties in the Netherlands approximates 12.7%. This implies a capacity loss in the medical workforce, a waste of educational resources and personal damage to individuals. The aim of our study was to investigate reasons for dropping out of residency and the relationship with medical work experience after medical school and prior to residency, which is common among Dutch graduates.

**Method:**

A questionnaire listing 28 reasons for drop-out was developed and tested. The questionnaire was sent in a nationwide survey to all residents who drop out between 1 September 2017 and 1 September 2019. The respondents were asked to indicate on a 5-point Likert scale, how they weighed reasons for drop-out. Factor analysis was applied to identify dominant factors.

**Results:**

The response rate was 39% (N=129; 99 females) representing all medical specialties. The factor structure of our measure showed 5 factors; high emotional job demands, lack of professional satisfaction, incompatible lifestyle, tensions in working relationships and disappointing career perspectives. Of the respondents 69 (54%) had prior clinical experience as a physician-not-in-training in the same medical specialty before residency. The factor “lack of professional satisfaction” weighed heavier for respondents without prior experience as a reason for drop-out.

**Conclusion:**

Of influence on dropping out of residency is the lack of a clear image of the responsibilities as a physician within the residency of choice, fuelled by limited prior experience after medical school and before residency. One third of dropouts confirmed that prior physician experience within the same specialty could have prevented dropping out or prevented choosing this specialty in the first place.

## Introduction

After medical school, most graduates start residency training, but some do not complete it, voluntarily or not. Terminating residency has significant impact, creating medical workforce capacity loss – populations must be served with an adequate mix of medical specialists – and often signalling personal tragedies [1], [2]. Terminating residency training or switching specialties is uncommon [3] but can cause damage to residents, psychologically as well as financially [2]. Students and graduates feel pressured to make the right choice at once and to avoid the stress of dropping out of residency [4]. This choice has a significant impact on career satisfaction and personal well-being in later life [1], [2], [5], [6], [7], [8]. Health and wellbeing of physicians have been found to relate positively to quality of care and patient satisfaction [2], [8], [9], but the prevalence of depression or depressive symptoms is high [10], [11], [12]. Insight into factors influencing drop-out from residency is limited. Most studies that provide valuable data on factors affecting drop-out report about one specialty, with a dominance of surgery [13], [14], [15]. In the US, a 2.6% attrition rate was found among 6,303 general surgical residents of 2007-2008, caused by a switch of specialty or true drop-out [16]. The authors found that the majority of attrition occurs early in residency; many residents re-enter in non-surgical residencies such as anaesthesiology, radiology and family medicine or commence a non-medical career [16]. Some studies investigated single factors such as gender [17], motivation rate [18], burn-out [19] or timing of drop-out [20]; one recent review and meta-analysis of studies among surgical residents reported that the majority of drop-out occurs after the first postgraduate year because of lifestyle-related issues [14]. 

In the Netherlands, almost all medical students follow a 6-year program, directly after secondary school. There is considerable time variation in the completion of medical school. Most students graduate after 6.5 to 7 years and at various moments during the calendar year. All eight schools offer ample elective space, specifically in the final year, which is called a “transitional year”, in which student are called “semi-physicians” and bear increased responsibility for patient care [21]. Graduation from medical school provides eligibility to receive a medical license, without further examinations, and to apply for a residency. 

Most graduates do not proceed directly to residency but take an interval period, up to multiple years, to gain clinical or research experience and so optimise their chances for selection for residency. Hospital wards, emergency departments and nursing homes offer many job opportunities for junior doctors – “physicians-not-in-training” (PNITs) as they are called.

Dutch graduates apply for residency positions in an open job market system [21], [22], [23], [24]. Throughout the year, approximately 2,400 graduates become eligible to start residency in a hospital residency or a non-hospital residency [25]. There is no national procedure to match graduates with residencies, as in North America [26], [27], [https://www.nrmp.org/]. 

In the Netherlands, about 175 residents quit every year before completion of postgraduate training (2013-2017) [28], [29]. The overall attrition rate is 12,7%, ranging between specialties from 4.9% to 24.4% [28], [29]. 

An unpublished Dutch longitudinal survey in 2009-2019 [30], [31], [32], [33] revealed mostly lifestyle related reasons for drop-out: high job pressure, long or irregular working hours, difficulty to combine residency with a family life, personal reasons, but also content of the educational program, desire to switch to another specialty, lack of personal coaching and a conflict at work [30], [31], [32], [33]. Another Dutch study among residents who terminated a hospital-residency included switching residents and found work-private balance, work pressure and disappointing content of the specialty as main reasons [34]; 65% chose a non-hospital residency as a career switch. Uncertainty about career choices is reflected in frequent adaptations of existing preferences, shortly before residency [32], [35], [36], [37], [38].

Our study aimed to identify reasons why trainees quit residency, across all specialties. In an earlier study we showed that the experience with responsibility for patient care as PNIT before residency is important to make a thoughtful career choice [37]. In the current study we looked especially into PNIT experiences of graduates who had dropped out. Our study aim was to provide insight into the dynamics of drop-out of residency and the significance of work experience as a PNIT before residency.

## Method

### Participants and procedure

At the time of the study 14,812 residents, 67% females with a mean age of 34 were active in the Netherlands. All residents are registered at the Dutch Medical Registration Council. The study population was extracted from the Dutch Medical Registration Council database and consisted of all residents (348) who prematurely terminated residency between September 1, 2017, September 1, 2019. 

An email including a link to the online survey, accompanied by a letter explaining the study purpose, was sent 19 September 2019, on our behalf by the Dutch Medical Registration Council. The research team had no access to email addresses or other personal information and the Medical Registration Council had no insight in the collected data. The study was approved by the Netherlands Association for Medical Education Ethical Review Board (#ERB197). The participants were informed that participation was voluntary, that confidentiality was secured and that non-participation would not be held against them. They could withdraw from the survey at any time without giving a reason. Written informed consent was obtained from all participants by answering a first survey question. Twelve days after the initial mailing, a reminder was sent. 

#### Instrument

As we did not find a suitable existing survey, we composed one (see attachment 1 ). We used a recommended seven-step process for survey design [39]. Interviews were held with three individuals who had dropped out of residency, which led to additions to our list of factors to represent the construct derived from the literature. Next, we developed items, using the vocabulary of the target population. For further content validation we selected three experts with methodological knowledge or research experience with the topic of drop-out for a panel to provide feedback on the draft survey. Next, one researcher (SQ), conducted three cognitive interviews to examine response process validity, i.e., assessing how prospective participants would interpret the survey items, using a think-aloud technique. This led to minor language edits. The themes included work experience prior to residency, preferences during the phase of career choice, current employment and desired employment, and a list of 28 potential reasons for dropping out of residency using a 5-point Likert-type scale (1=not a reason to quit residency to 5=important reason to quit residency) and demographic information, including gender, age, specialty, part-time or fulltime residency program, drop-out year. Finally, the survey was pilot tested in SurveyMonkey^®^ [https://www.surveymonkey.com/] with the research team and two frequent Survey Monkey users (no physicians), not leading to further changes.

#### Analysis

We divided our respondents into 2 groups based on hospital and non-hospital specialties, because of different residency characteristics such as training period, working hours and working conditions. Differences between subgroups were calculated using crosstabs and Chi-square tests. Subgroup analyses were performed based on gender, specialty, part-time/fulltime and previous employment (PNIT-experience) in the same specialty. To determine the underlying dimensions, the researchers performed an exploratory factor analysis on the 28 reasons for dropping out of residency, using principal component analysis with varimax rotation using IBM SPSS^®^ software version 26.

The answers to the open-ended questions were analysed by researchers SQ, MdR and LW. All data was set in an excel template and response categories were identified. The individual responses were organised and themes were noted. Any discrepancies in data interpretations were resolved in team discussions. 

## Results

### Study population characteristics

The email was sent to 348 graduates (72% females) who had prematurely left specialty training. Ten unusable email addresses and 3 persons not wishing to be included left an eligible group of 335. Among the 145 respondents who participated, 16 left incomplete responses and were excluded. With 129, the response rate was 39%, including 99 (77%) females.

The mean age was 34.8. Of the respondents, 55% (63 females and 8 males), had a part-time contract, which is a legal right in the Netherlands down to 80% fulltime, while 45% (36 females and 22 males) were fulltime.

For 86% of the respondents (111), the specialty which they had left was first choice; for 9% (12) second choice and for 5% (6) third choice. Most, 86% (111), had left residency in the first three years of the training period; 79% (102) had made their own decision to discontinue, in 10% (13) the decision was forced by the program and in 10% (14) it was a joint decision. Most respondents currently work as a PNIT or have started a different residency; a small group is not employed as a physician. The study population characteristics are summarised in table 1 (Tab. 1). Some respondents did not answer all questions, the total response group is noted.

#### Distribution of response across specialties

The Advisory Committee on Medical Manpower Planning calculated, based on average drop-out rates 2004-2019, the percentage of residents expected to complete training (see table 2 (Tab. 2), column 2). We related the numbers of survey respondents for each specialty program to these data (see table 2 (Tab. 2), column 3). 

#### Experience related to the specialty of drop-out

Some respondents had prior experience in the same specialty. Of the 127 respondents who provided information about prior work experience, 13 (10%) reported having had no experience in the specialty of their residency; 57 (45%) had an elective subinternship in medical school in the same specialty; 69 (54%) had PNIT experience in the same specialty and 16 (13%) had both; 29 (23%) had experience as PhD student, i.e. research experience, before residency. 

The respondents with prior PNIT-experience can be categorised into four groups: 1-12 months (N=11), 13-24 months of experience (N=28) more than 24 months of experience (N=18). Another 12 respondents did have work experience but did not indicate the duration.

#### Factors affecting drop-out 

The factor structure of our measure of drop-out reasons showed five factors (see table 3 (Tab. 3)). Only the items with a factor contribution of 0.36 or higher are included

Factor 1, “high emotional job demands” (independent of time) with an α of 0.734, was comprised of 3 items; energy-consuming work, I had (symptoms of) a burnout, and emotionally too heavy for me.

Factor 2, “lack of professional satisfaction” (independent of effort, time and expectations) with an α of 0.741, comprised 7 items; the residency did not meet the expectations suggested by advance information, insufficient satisfaction with the nature of the work, not being able to really care/mean something to the patient, professional autonomy limited by rules/limited autonomy, performing badly, too much pressure for (medical) production and less for personal development, and the work being different than expected.

Factor 3, “incompatible lifestyle” with an α of 0.738, comprised 7 items; work-life imbalance, lifestyle incongruence, high volume of duty hours, it was physically too heavy for me, financial matters/problems, personal or family related reason, and there was no possibility to work parttime.

Factor 4, “tensions in working relationships” with an a of 0.725, included 5 items; interpersonal problems (colleagues/team), forced by others to quit, inability to share personal concerns with someone, could not cooperate/work with superior, and suffered from inappropriate relational transgressions at work.

Factor 5, “disappointing career perspectives” with an α of 0.562, had 5 items; status of the specialty, work setting/type of practice, limited career perspective within this specialty, opinions of others about the specialty influenced me, and impact of disciplinary or legal procedures.

One item “I became interested in another specialty” did not show factor loadings on any of the five factors. 

Next, we contrasted for the five factors those respondents who had prior clinical experience in the specialty as a junior doctor (PNIT) with those who did not have that experience. “Lack of professional satisfaction” was significantly more important as a factor for drop-out for those without previous experience (mean 2.29, SD 0.77) than for those with previous experience within the same specialty (mean 1.91, SD 0.73, significant 2-tailed *P* value 0.005) (see table 4 (Tab. 4)).

#### Preventing drop-out 

We also asked what could have prevented them from quitting residency. This was an open question and answered by 42 respondents. The responses were categorised in four themes, besides a category of irrelevant personal and miscellaneous reasons:

1. more orientation about specialties, mainly through experience as a PNIT (n=13),
2. more detailed information about the structure of the residency (n=9),
3. personal coaching (n=6), and point
4. safer learning climate (n=2).

Next, we also asked what could have supported them not to start with this residency. This was also an open question and answered by 46 respondents. We found three themes:

1. better orientation about the specialties, mainly through experience as a PNIT (n=21),
2. information about how to combine residency with raising a family (n=5), and
3. detailed information about the workload and type of work as a resident (n=5).

## Discussion

We conducted a nationwide survey among residents who had dropped out of specialty training, to obtain insight into the factors involved in terminating residency and to investigate whether these reasons differ among those having prior PNIT experience or not. Our study supports earlier findings that drop-out most often takes place in the first three years of residency [20], [34], [40].

We found five themes:

1. high emotional job demands,
2. lack of professional satisfaction,
3. incompatible lifestyle,
4. tensions in working relationships and
5. disappointing career perspectives.

These results are in line with previous research of drop-out in the hospital specialties [20], [30], [31], [32], [33], [34], [41]. Our study indicates that these factors are of influence for all graduates who drop out. A prominent observation is that 46% of the respondents had not had prior PNIT experience with the responsibilities and daily work of the specialty of choice. Previous research found a higher percentage of PNIT experience among hospital residents [34]. Related to the main themes pointed out as reasons for drop-out by the respondents this may indicate that PNIT experience is pivotal before starting as a resident. Of all Dutch graduates it is estimated that 64% has prior experience as a physician in the specialty of their career choice [33]. PNIT experience as a family physician is not possible in the Netherlands, and some of our respondents dropped out of this specialty. Our study is new, and further validation of the five indicated factors of influence would be useful.

We consider the factor “external pressure”, i.e. pressure caused by someone or something outside the resident, of major concern, because of the relation with burn-out and the effect on the physical and psychological well-being of physician's [10], [11], [12]. Taking care of the physician’s well-being is pivotal for our future healthcare system [9], [12], [42], [43]. Therefore, more attention to these lifestyle-related issues is crucial. The factor “financial matters/problems” is not likely to impact the considerations to terminate residency in the Netherlands; Dutch residents usually have a reasonable salary and limited debts after medical education. 

This study strengthens previous findings [14], [34], that prior experience within the same specialty is useful to generate a realistic image of the demands of being a resident and thus would prevent choosing this specialty and consequently prevent dropping out [37], [44], [45]. We found that the factor “nature of the work” weighs heavier (i.e., shows a higher score) for those without prior experience with the same specialty than for those with this experience as a reason for drop-out (see table 3 (Tab. 3)). Up to a third of the respondents suggests that better orientation and more PNIT experience could have prevented drop-out. In the Netherlands physicians can work as a PNIT and experience the specialty prior to residency. Those with realistic expectations of the demands of residency and life as a resident may be more likely to complete their residency [45]. It means long days, hard working, high performance and act as a licensed physician. These findings support our hypothesis that PNIT experience in the same specialty is relevant for making a valid career choice. It is likely that graduates would benefit from having a PNIT experience to explore the full scope of a specialty and inform themselves about the structure, type of work and the demands of the residency before making their definite career choice. That prior patient care experiences could prevent drop-out was also suggested by Khoushal [41] and Bustraan [34]. In an earlier study about factors determining medical career preference we found that personal needs satisfaction (including lifestyle and work-life balance considerations), perceived specialty characteristics (including intellectual satisfaction, types of team and colleagues, types of patients, nature of the work) were critical [36]. These, if not satisfied, may likely also cause drop-out.

Mentoring could help prevent burn out, help trainees cope with high work load and navigate the balance between work and private life, as these seem important reasons for drop-out. Dutch Family Medicine and Psychiatry residencies have now integrated coaching in their programs [46], [47]. Another approach that has been applied is peer-supervision among residents [46], [47]. 

Next, to support structures to assist residents individually in coping with the challenges or residency training, the training environment itself may be subject to scrutiny. Health care, with or without training, is an increasingly demanding working environment, that appears to be less and less attractive for young physicians to work in [48], [49], [50]. Contextual adaptations may be necessary to prevent increasing numbers of residents choosing to leave this work environment. Also, residency program directors should attempt to increase the compatibility of residency with personal life and to better orient and support residents. 

### Limitations

This study has limitations. This was a retrospective study with residents who quitted residency within two prior years. It is possible that those respondents who left residency long ago may have forgotten details; a selective memory could have affected their answers. The survey was sent to all residents who dropped out during this period. However, the survey was sent anonymously, and we could not send personal reminders. While our sample was a fair representation of the population, seven respondents exceeded the age of 45. We initially excluded them from our sample, but as this did not lead to essential differences in the results we included them. Our study findings could have been affected by a somewhat larger proportion of women in our sample: 76% in our study and 72% in the study population group vs 67% in the group of Dutch residents. There was also a larger proportion of part-time respondents: 55% in our sample and 38% in the reference group vs 47% in the group of all Dutch residents. With these points in mind, we should be careful with the interpretation of the differences between the subgroups. 

## Conclusion

The results of this study support the notion that, to make an adequate medical career choice, graduates must have a realistic insight of their preferred specialty. Graduates may likely benefit from PNIT experience in their preferred specialty before making their definite career choice. Furthermore, residents may benefit from support focused on how to deal with lifestyle related issues, professional and personal development during their residency. 

## Acknowledgements

For this study, we gratefully collaborated with the Medical Registration Council (RGS) of the Royal Dutch Medical Association (KNMG). The Medical Registration Council has a complete documentation of all Dutch graduates, residents and specialists [https://www.knmg.nl/opleiding-herregistratie-carriere/rgs/over-de-rgs.htm]. The Council periodically tests whether doctors and study programs comply with the rules of the Medical Specialties Board. The Medical Registration Council registers contain information about education programs, duration, qualifications, years of practice and drop-out rates. 

The authors like to further thank Michiel Wesseling, PhD, former director of the Medical Registration Council, Utrecht and Bram Peters, administrator of the Medical Registration Council for their support in extracting the data and sending the survey; Eugene Custers at the time senior scientist at the Center for Research and Development of Education, Utrecht and Kirsten Dijkhuizen PhD student at Leiden University Medical Center, for their support with developing the survey; Heleen Pennings, PhD, for her support with the factor analysis; and Victor Slenter, director at Advisory Committee on Medical Manpower planning, Utrecht, for his expertise in developing the survey and for his suggestions for analyzing the data.

## Competing interests

The authors declare that they have no competing interests. 

## Supplementary Material

Survey questions about reasons why resident prematurely left their residency

## Figures and Tables

**Table 1:**
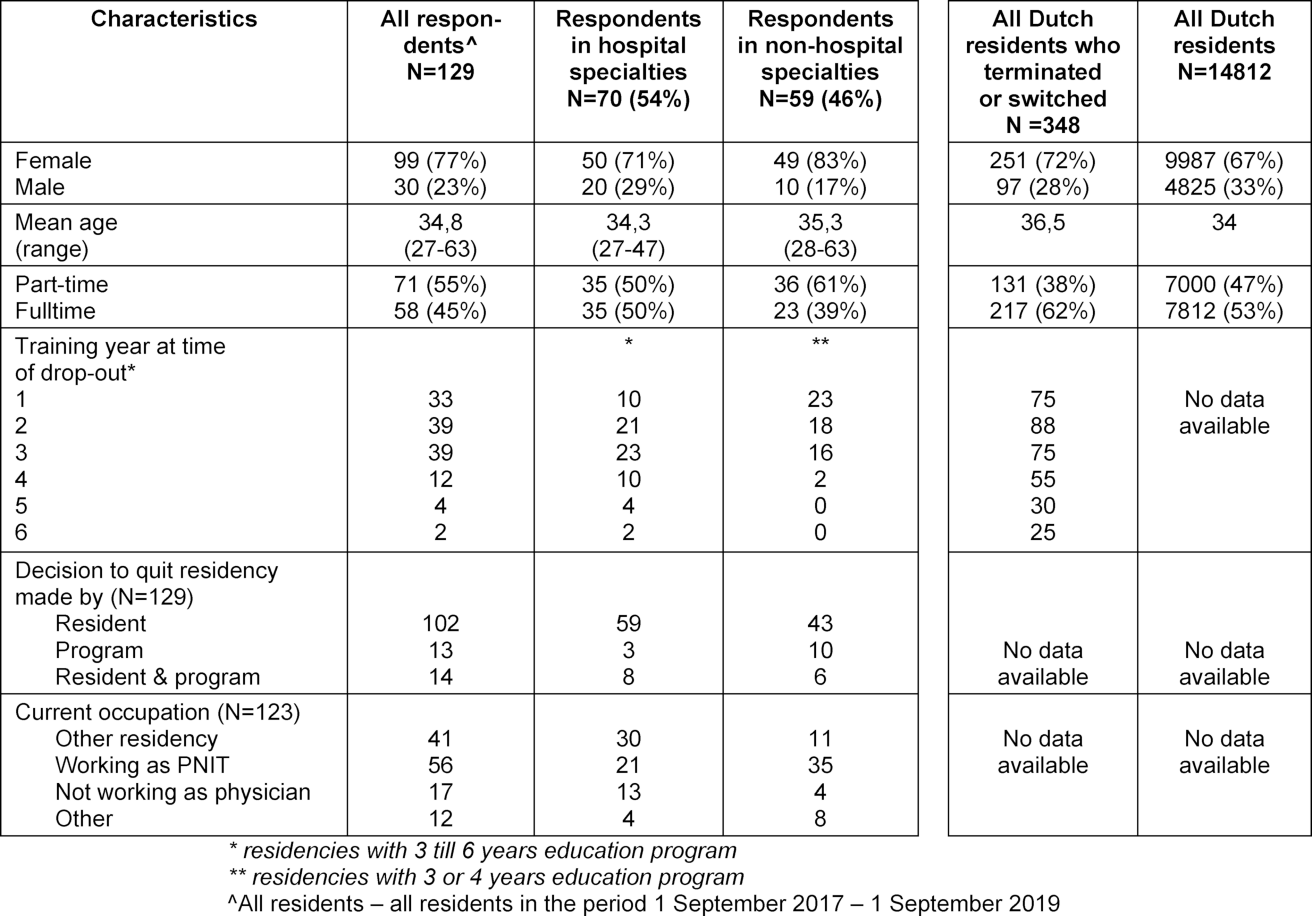
Overview of study population characteristics

**Table 2:**
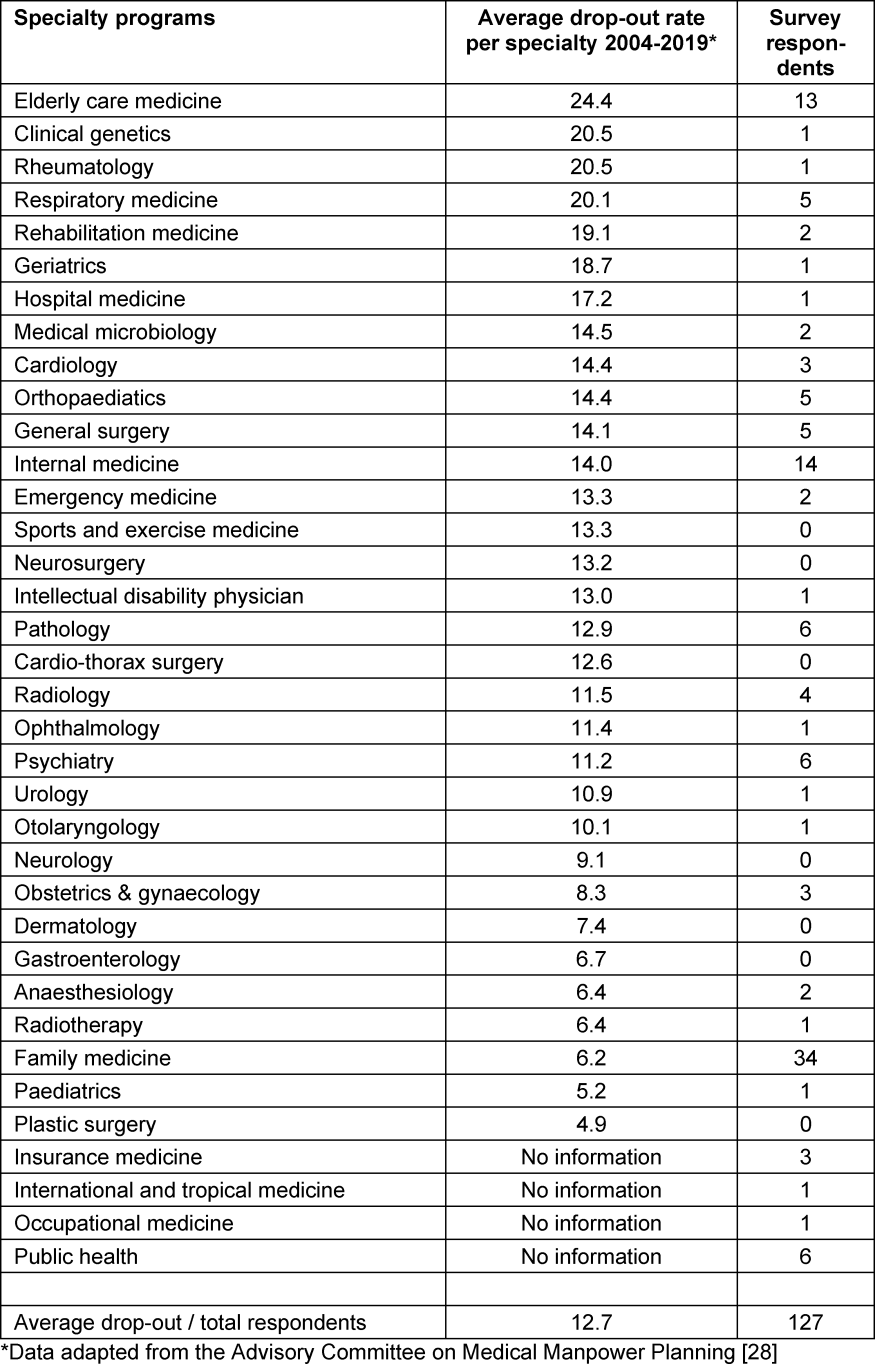
Overview of response across specialties

**Table 3:**
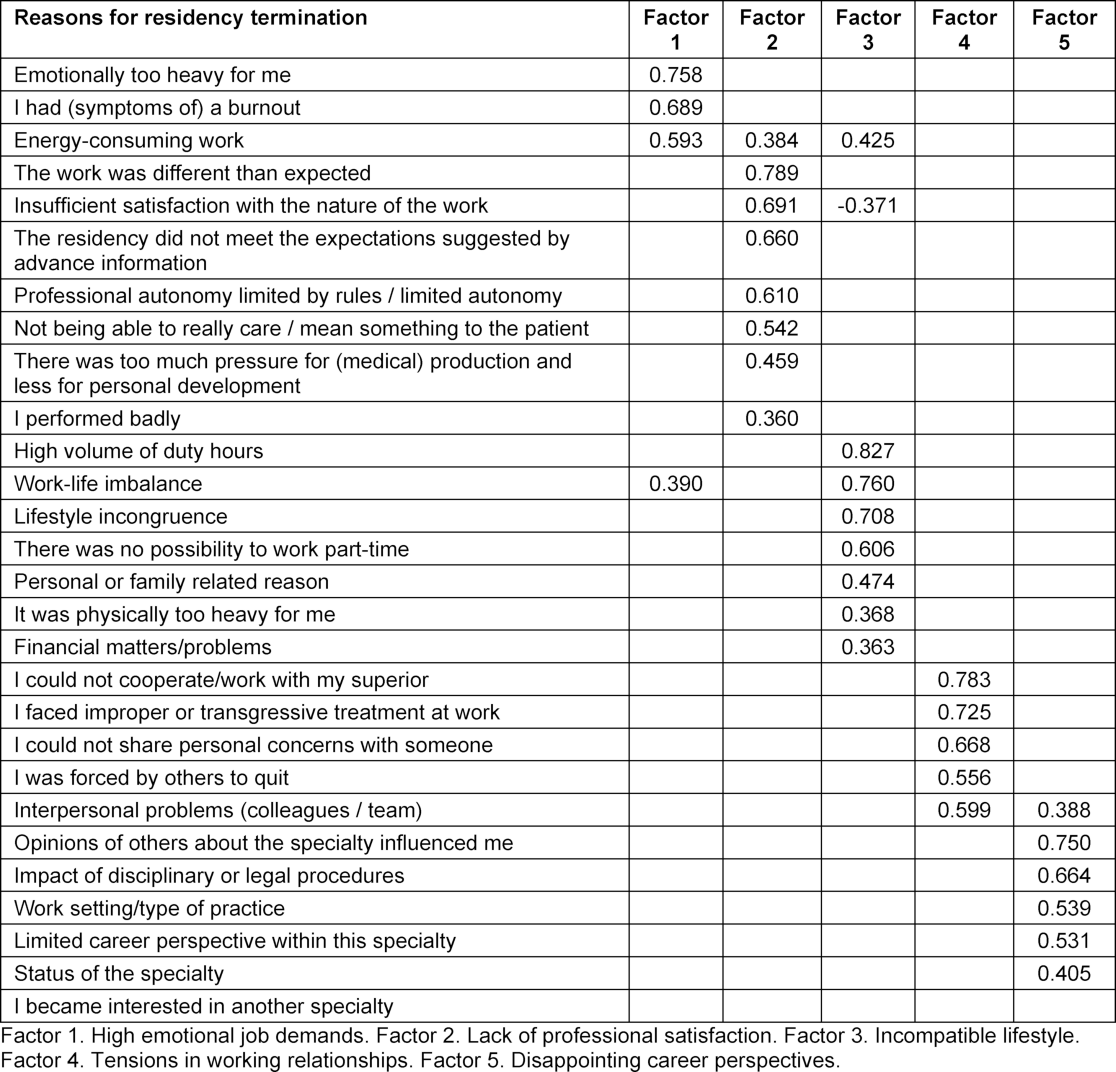
Factor loadings of the questionnaire items, 28 reasons for residency termination

**Table 4:**
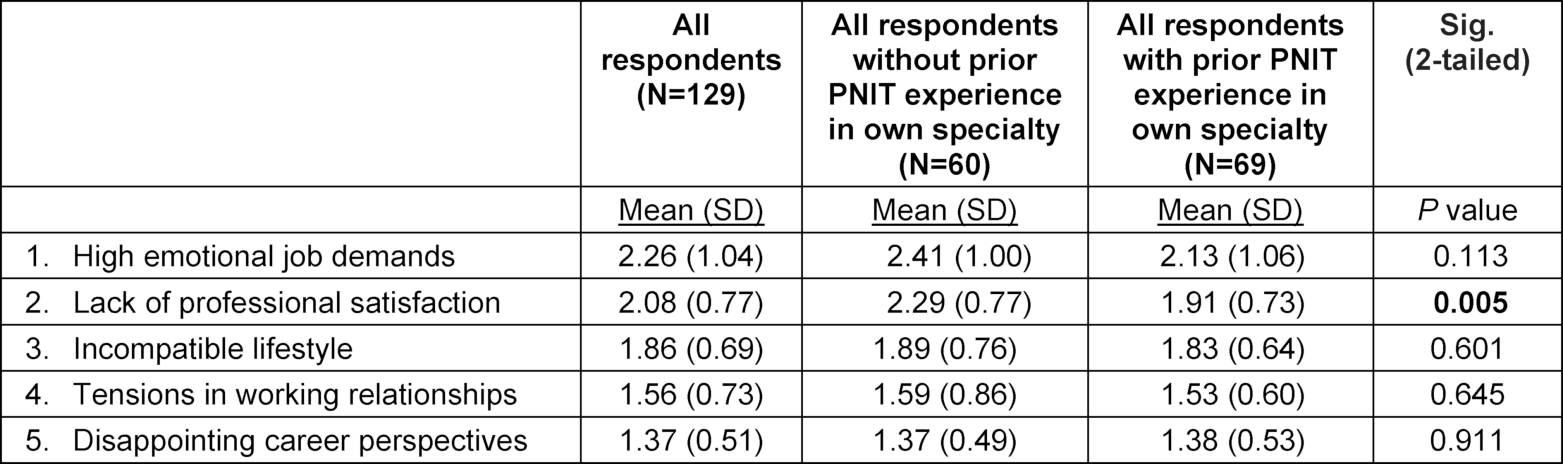
Reasons for residency termination, based on factor ¬analysis of 28 survey items, and differences for residents with and without focused PNIT experience
