# Supplementary material for: Some residents drop out of specialty training. How important is prior clinical experience? A survey among residents in the Netherlands
Source: GMS J Med Educ. 2023 Feb 15;40(1):Doc5. doi: 10.3205/zma001587 (PMC10010763; doi:10.3205/zma001587)
Supplement: Survey questions about reasons why resident prematurely left their residency [file JME-40-5-s-001.pdf]

**Attachment 1: Survey questions about reasons why resident prematurely left their residency**

1. Informed consent *(check box)*
2. What is your age? *(open question)*
3. What is your gender? *(male/female)*
4. How was your personal situation when you quit residency? *(alone without children, alone with children, relation and living apart, relation and without children, relation and with children, other.*
5. Did you had children when you quit residency? *(0,1, 2 or more then 2).*
6. What was your contract during residency? *(part-time/fulltime).*
7. Which residency did you quit? *(open question)*
8. Was this residency your first, second, third (or more) choice? *(1st, 2nd or 3rd or more).*
9. When did you graduate from medical school? *(dd/mm/yyyy).*
10. When did you start residency? *(dd/mm/yyyy).*
11. When did you quit residency? *(dd/mm/yyyy).*
12. Which year of residency did you quit? *(year 1, year 2, year 3, year 4, year 5, year 6).*
13. Was it your choice or the supervisor's choice to quit residency? *(own choice, supervisor's choice, made choice together)*
14. What was the main reason to quit residency? *(open question).*
15. Can you indicate of the following factors how they influenced the decision to quit residency? *(28 specialty related and personal items on a 5-point Likert scale to indicate relevance).*
16. Are there other factors that influenced the choice to quit residency? *(open question).*
17. What could have persuaded you to continue residency? *(open question).*
18. Did you have any work-related experience in the specialty of your residency before you started? *(no work experience, work experience as final year student, work experience as graduate, work experience as PhD, work experience as other option).*
19. Can you describe this work experience? *(open question).*
20. Which information, experience, or support before starting residency could have prevented that you had quitted residency? *(open question).*
21. Which information, experience, or support before starting residency could have prevented that you had started this residency? *(open question).*
22. What is your current job position? *(other residency, other job as graduate, other job not as graduate, orientation, other).*
23. Do you have any comments or suggestions regarding this survey? *(open question).*

Attachment 1 to Querido SJ, de Rond ME, Wigersma L, ten Cate O. *Some residents drop out of specialty training. How important is prior clinical experience? A survey among residents in the Netherlands*. GMS J Med Educ. 2023;40(1):Doc5. DOI: 10.3205/zma001587
